# Analysis of Pollen Allergens in Lily by Transcriptome and Proteome Data

**DOI:** 10.3390/ijms20235892

**Published:** 2019-11-24

**Authors:** Jingxian Feng, Ze Wu, Xueqian Wang, Yaming Zhang, Nianjun Teng

**Affiliations:** 1 College of Horticulture, Nanjing Agricultural University, Nanjing 210095, China; 2017104105@njau.edu.cn (J.F.); wuze@njau.edu.cn (Z.W.); 2016104112@njau.edu.cn (X.W.); 2017804184@njau.edu.cn (Y.Z.); 2 Key Laboratory of Landscaping Agriculture, Ministry of Agriculture and Rural Affairs, Nanjing 210095, China; 3 Baguazhou Science and Technology Innovation Center of Modern Horticulture Industry, Nanjing 210043, China

**Keywords:** Lily, pollen, transcriptome, proteome, allergen

## Abstract

The lily (*Lilium* spp.) anther contains a lot of pollen. It is not known if lily pollen contains allergens, and therefore screening pollen allergy-related proteins and genes is necessary. The pollen development period of lily ‘Siberia’ was determined by microscope observation. Early mononuclear microspores and mature pollens were used as sequencing materials. The analysis of the pollen transcriptome identified differentially expressed genes (DEGs), e.g., *Profilin*, *Phl p 7 (Polcalcin)*, *Ole e 1*, and *Phl p 11*, which are associated with pollen allergens. The proteome analysis positively verified a significant increase in pollen allergenic protein content. The expression levels of LoProfiilin and LoPolcalcin, annotated as allergen proteins, gradually increased in mature pollen. LoProfiilin and LoPolcalcin were cloned and their open reading frame lengths were 396 bp and 246 bp, which encoded 131 and 81 amino acids, respectively. Amino acid sequence and structure alignment indicated that the protein sequences of LoProfilin and LoPolcalcin were highly conserved. Subcellular localization analysis showed that LoProfilin protein was localized in the cell cytoplasm and nucleus. LoProfilin and LoPolcalcin were highly expressed in mature pollen at the transcriptional and protein levels. A tertiary structure prediction analysis identified LoProfilin and LoPolcalcin as potential allergens in lily pollen.

## 1. Introduction

Lily (*Lilium* spp.) is a perennial herbaceous bulbous flower of the Liliaceae, which has ornamental, medicinal, and edible functions [1,2]. The lily is also one of the world’s main cut flowers due to its beautiful color and pleasant fragrance [3]. However, the anthers of lily are huge and contain a large amount of pollen; therefore, when the flowers open, pollen pollution will cause many problems, such as clothing contamination, and could also affect the health of people [4,5]. Pollen is one of the main factors determining the occurrence of seasonal allergies. Unlike food allergies, pollen allergy is often unavoidable [6]. During the period of flower opening, pollen grains are released into the air to form biological aerosols; thus, individuals are inevitably exposed to pollen [7]. Pollen pollution seriously affects the quality of life for people susceptible to allergies. However, there have been few studies of the allergic proteins in ornamental plants, especially lily.

The outer wall of pollen is mainly composed of sporopollenin, lipids, proteins, and other substances, while various proteins and hydrophobic lipids are also present in the pollen voids and grooves [8]. The allergenic proteins on the surface of the pollen wall may cause allergies in susceptible individuals [9]. Many studies have reported dozens of proteins distributed on the surface of pollen, and pollen allergens are the main pollen surface proteins, including the profilin and polcalcin family proteins [10,11]. They are called panallergen because the same family of proteins have a common antigen reaction cluster, and they can cause a wide range of cross-reactions [12,13]. Plant profilin was identified as an allergen in 1991 [14]. In Arabidopsis, the profilin gene family is divided into two major classes: Vegetative profilins and reproductive profilins [1]. *AtPRF4* and *AtPRF5* are mainly expressed in mature pollen [15]. A polcalcin family protein, Phl p 7, is a pollen-specific polcalcin allergen in Timothy grass [16]. Phl p 7 and polcalcin homologues are present in the pollen of many species, and most are positioned on the pollen wall [17]. The majority of allergen studies have focused on the biochemical characteristics, and there have been only a few studies of the production of allergic proteins.

In the past decade, many common allergic pollens belonging to different species have been reported in clinics. Researchers have isolated and identified various pollen allergens, such as *Humulus scandens* [18], *Artemisia vulgaris* [19], and *Ambrosia artemisiifolia* [20]. The identification and purification of pollen allergens is of great significance for pollen allergy problems. According to the structure of the plant allergic protein, plant allergic proteins can be divided into four major families: The profilin, prolamin, cupin, and Bet v 1 protein families [21]. Proteins of the same family share a common domain and are relatively conservative in structure. Therefore, the common allergen proteins can be identified in a variety of plants [14,22]. With the developments in molecular biology technology, the identification of allergens has become increasingly comprehensive. In particular, the combination of transcriptome, proteome, and immunoblotting analyses has been very helpful for the identification of common allergens in species without any reference genome sequence. This method has been successfully applied to important allergens, such as aphids and ragweed [23].

To screen and predict lily allergens, we compared gene and protein differences between mononuclear microspores and mature pollen through transcriptomes and proteomics. A profilin and a polcalcin proteins, were specially expressed and cloned from lily pollen. This laid the foundation for our research on lily pollen allergens.

## 2. Results

### 2.1. Morphological and Cytological Characterization of Lily

**Figure 1:**
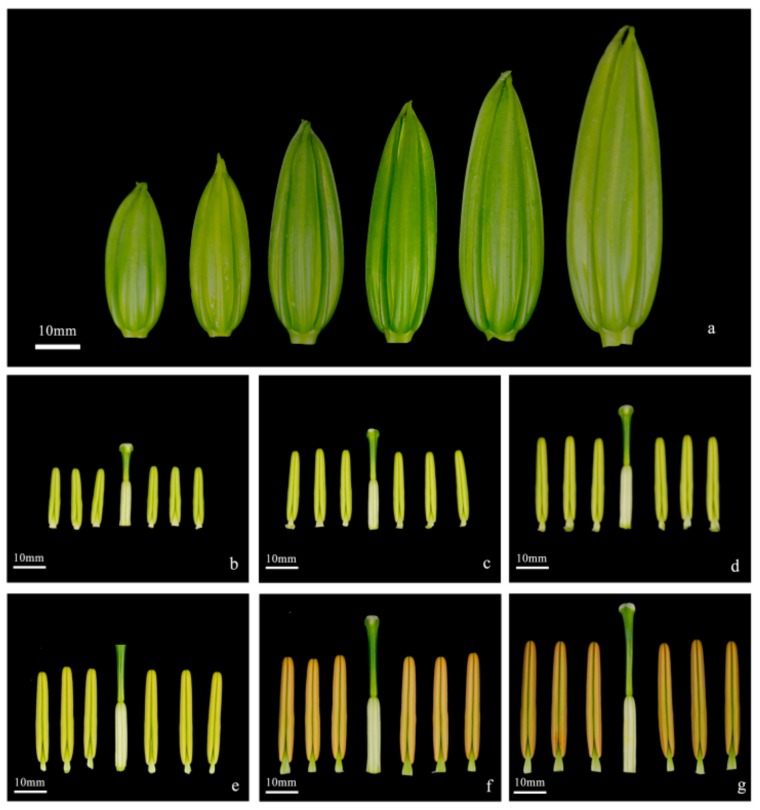
Morphological characteristics of *Lilium* oriental hybrid ‘Siberia’. (**a**) Flower buds of six periods. (**b**–**g**) Pistil and six anthers in different stages. (**b**) sporulation stage; (**c**) mother cell stage; (**d**) meiosis stage; (**e**) tetrad stage; (**f**) early mononuclear stage; (**g**) mature pollen stage. Bar = 10 mm.

**Figure 2:**
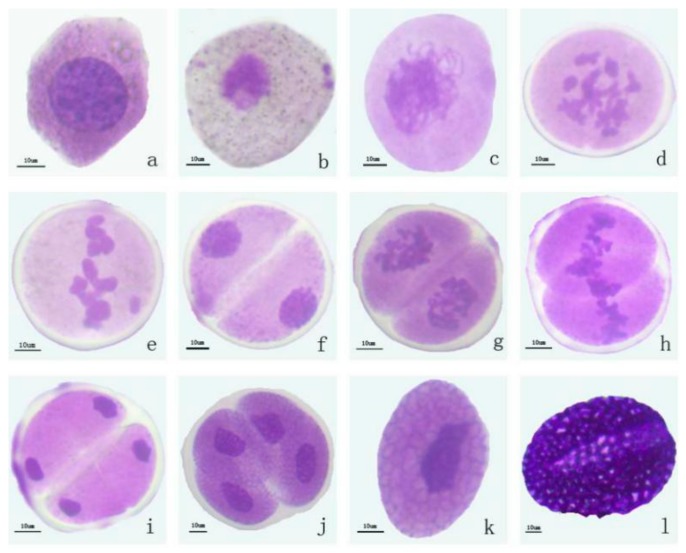
Lily pollen development period. (**a**) Sporulation stage of *Lilium* oriental hybrid ‘Siberia’. (**b**) Mother cell stage of *Lilium* oriental hybrid ‘Siberia’. (**c**–**i**) Meiosis stage of *Lilium* oriental hybrid ‘Siberia’. (**j**) Tetrad stage of *Lilium* oriental hybrid ‘Siberia’. (**k**) Early mononuclear stage of *Lilium* oriental hybrid ‘Siberia’. (**l**) Mature pollen stage of *Lilium* oriental hybrid ‘Siberia’. Bar = 10 mm.

According to the pollen morphology at different development stages of the flower bud, the period of pollen development could be roughly divided into six periods: The sporulation, mother cell, meiosis, tetrad, early mononuclear, and mature pollen stages. The flower buds, anthers, pistil size, anther color, and pollen color in the different periods were observed and recorded (Table 1, Figure 1), for the preparation of subsequent sampling. It was found that the pollen development was positively correlated with the size of anther. The size of the anther in the tetrad stage was 29–32 mm, and the color of the anthers was light yellow. The pollen development in different periods is shown in Figure 2. In the early mononuclear stage (Si-1), the pollen wall was clearly visible, but the texture was not obvious. In the mature pollen stage (Si-2), the pollen wall was completely formed, and the germination groove could be observed (Figure 2).

### 2.2. Transcriptome Sequencing and Transcript Assembly

Six cDNA libraries (including three biological replicates) from ‘Siberia’ at the Si-1 and Si-2 stages of pollen development were subjected to Illumina sequencing. After assembling and cleaning the data, we obtained 62,398,416 (9.36 Gb) clean reads from three Si-1 libraries. The clean reads ratio was 95.03%, and the Q20, Q30, and GC percentages were 97.11%, 92.56%, and 50.81%, respectively. We obtained (9.68 Gb) clean reads from three Si-2 libraries. The clean reads ratio was 96.01%, and the Q20, Q30, and GC were 97.32%, 92.85%, and 51.72%, respectively. After splicing all clean reads, the number of transcripts and unigenes were 138,564 and 102,490, respectively, and the average length of a unigene was 610 bp. The results are shown in Table 2.

### 2.3. Gene Function Annotation and Analysis of Differentially Expressed Genes (DEGs)

To obtain comprehensive information regarding gene function, we performed a gene function annotation based on the seven databases (Nr, Nt, Pfam, KOG, Swiss-prot, KEGG, and GO). A total of 30,347, 11,752, 11,094, 23,357, 24,513, 24,513, and 7326 unigenes were annotated, respectively. Through comparison with the Nr database annotation, the similarity between the gene sequences of the species and the gene sequence of the related species could be obtained. The functional information of the gene sequence was also obtained. Annotated results through the Nr library, statistics, and mapping of species distribution found that 21.4%, 17.8%, 7.0%, and 6.4% unigenes were closely matched with *Elaeis guineensis*, *Phoenix dactylifera*, *Asparagus officinalis*, and *Musa acuminata*, respectively (Figure S1). Like lilies, these plant species are monocots.

**Figure 3:**
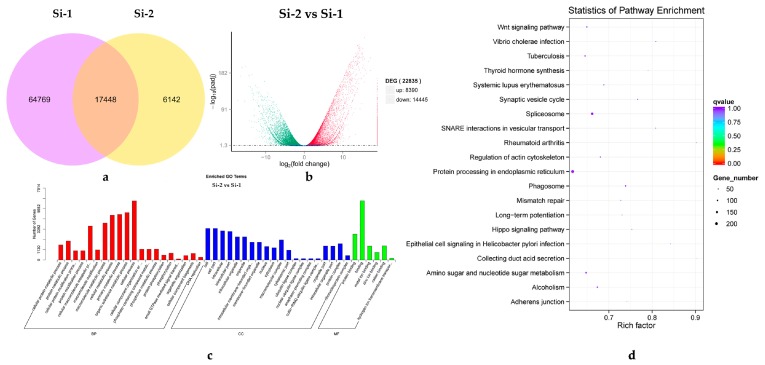
Differentially expressed gene (DEG) detection map. (**a**) Venn diagram of DEGs. The purple part indicates the genes contained in Si-1; The yellow part indicates the genes contained in Si-2; The overlapping part indicates the genes shared by Si-1 and Si-2. (**b**) Volcano map of the DEGs. The abscissa represents the difference in the flod change in gene expression between the two periods. The ordinate indicates the degree of significance in the difference expression between the two periods. Up-regulated DEGs are represented by green dots, down-regulated DEGs are represented by red dots, and the blue dots represent no significant change in genes. (**c**) GO (Gene ontology) classification histogram of the DEGs. The abscissa represents different GOterms, and the ordinate represents the number of DEGs in each term. BP represents biological process, CC represents the cellular component, and MF represents the molecular function. (**d**) Bubble chart of the DEGs enrichment pathway. The abscissa represents the enrichment factor in each pathway and the ordinate represents the different pathways. The colors from red to blue indicate that the q value is getting bigger and bigger. The size of the point represents the number of DEGs.

The DEGs from two development periods of pollen were analyzed. Difference analysis was conducted using readcount data (after standardization by DESeq). The DEGs were selected based on their possible function and fold change in expression level in the Nr annotation database, with a screening threshold of padj <0.05. Venn diagrams were used to show the common genes and unique genes in each period (Figure 3a). There were more gene expressions in the Si-1 early mononuclear stage, with physiological activity likely during this period. A total of 22,835 DEGs were screened, of which 8390 were up-regulated and 14,445 were down-regulated. An analysis of the DEGs at different stages of pollen development indicated that there were significantly more down-regulated genes than up-regulated genes, and a volcano map was used to represent the DEG distribution (Figure 3b).

After the GO assessment of the DEGs, 11,305 successfully annotated DEGs were classified. They could be divided into three major categories of biological process, cellular component, and molecular function. The biological process category contained 20 terms, of which the major terms were ‘cellular process’, ‘organic substance metabolic process’, and ‘primary metabolic process’. The cellular component category contained 20 terms, with ‘cell’ and ‘cell part’ being the main terms. The molecular function category contained six subcategories, including ‘binding’ and ‘protein binding’. The classification results were shown in a GO classification map (Figure 3c). The top 20 DEGs enrichment pathway was represented by a bubble chart (Figure 3d).

**Figure 4:**
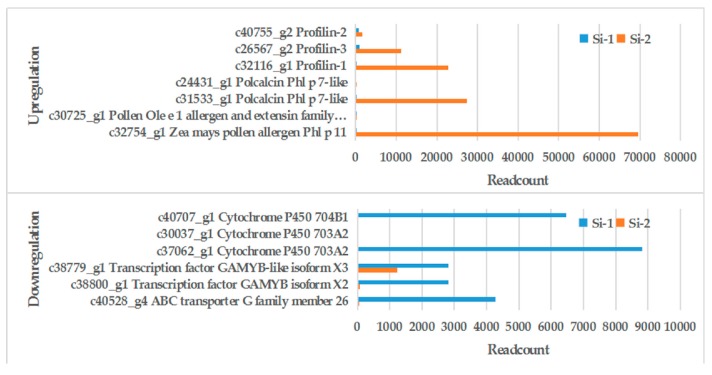
Histogram of differentially expressed genes (DEGs). Blue column stands for Si-1, red column stands for Si-2. DEGs expression were expressed by readcount.

Seven DEGs related to allergies were found, then three distinct profilins (c32116_g1, c26567_g2, c40755_g2), two polcalcins (c31533_g1, c24431_g1), and two Ole e 1-related proteins (c32754_g1, c30725_g1) sequences were characterized. The expression of these genes in the mature pollen stage was significantly higher than in the early mononuclear stage, especially c32116_g1 (*profilin*) and c31533_g1 (*polcalcin*). Histograms were produced to show their expression at different times (Figure 4). The pollen wall was observed in the early mononuclear stage; in this stage, a number of genes related to pollen wall synthesis were found. During the process of pollen outer wall formation, ABC transporter G family member 26, transcription factor GAMYB, and cytochrome P450 703A2 and 704B1 were associated with pollen wall synthesis [24,25,26,27]. They were highly expressed in the early mononuclear stage, indicating frequent physiological activity associated with the formation of the pollen wall in this stage. With the formation of the pollen outer wall, the content of many outer wall proteins increased. This indicates that the transcriptome data were basically consistent with the normal expression of the genes. The DEGs information is shown in Table 3.

**Figure 5:**
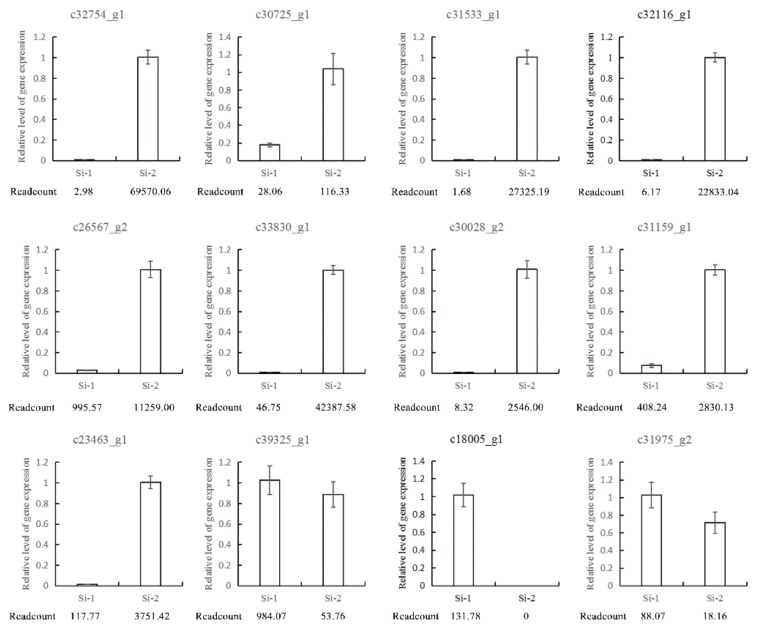
Validation of RNA-seq results by a quantitative real-time polymerase chain reaction (qRT-PCR). RNA-seq values are the readcount of two libraries with three biological replicates and three technical replicates. The qRT-PCR values were determined via qPCR using the –ΔΔCt values.

To confirm the quality of the RNA-Seq libraries, 12 DEGs were randomly selected for a qRT-PCR analysis at the two development stages, with readcount indicating the abundance in the sequencing data of the transcriptome libraries. These DEGs showed the same tendency of altered expression as in the RNA-Seq results (Figure 5).

### 2.4. Protein Function Annotation and DEPs Analysis

From the quantitative proteome analysis based on a tandem mass tag (TMT), we obtained 10,965 proteins for two periods. We performed a protein function annotation based on the GO, KEGG, COGm and IPR databases and annotated 3712 (33.85%), 10,636 (97.00%), 4844 (44.18%), and 8911 (81.27%) proteins, respectively (Figure S2a). The main terms included in the biological process category of the GO analysis were ‘oxidation-reduction process’ (408), ‘protein phosphorylation’ (251), and ‘metabolic process’ (213). The main terms in the cellular component category were ‘integral component of the membrane’ (322) and ‘membrane’ (252). The main terms in the molecular function category were ‘protein binding’ (966) and ‘ATP binding’ (738). The protein functions were classified through a COG annotation and divided into 25 groups, with the larger ones being ‘translation, ribosomal structure and biogenesis’ (665, 13.73%), ‘general function prediction only’ (648, 13.38%), and ‘posttranslational modification, protein turnover, chaperones’ (564, 11.64%), while the smaller ones included ‘extracellular structures’ (4, 0.08%) and ‘mobilome, prophages, transposons’ (5, 0.10%). Through a KOG annotation, the proteins were divided into 26 groups, with the three largest groups being ‘translation, ribosomal structure and biogenesis’ (665), ‘general function prediction only’ (648), and ‘post translational modification, protein turnover, chaperones’ (564).

A total of 2328 DEPs were screened. When FC ≥ 2.0 and the *p*-value ≤ 0.05, the protein expression was up-regulated expression, and the number of up-regulated proteins was 886. When FC ≤ 0.50 and the *p*-value ≤ 0.05, the protein expression was down-regulated, and the number of down-regulated proteins was 1442. Through a GO enrichment analysis, the DEPs were enriched to different terms depending on their function (Figure S2b). The main enrichment terms of DEGs in the biological process category were ‘metabolic process’, ‘organic substance metabolic process’, and ‘primary metabolic process’. Most of the DEGs performed functions associated with metabolic processes. The main terms in the cellular component category were ‘intrinsic component of membrane’, ‘intracellular non-membrane-bounded organelle’, and ‘integral component of membrane’. The proteins associated with the membrane component underwent large changes between the mature and early mononuclear stages. In the ‘nucleus’ term, a large number of DEPs were down-regulated, indicating that nuclear proteins also changed significantly between the early mononuclear and mature stages. The main term in the molecular function category was ‘catalytic activity’, and various catalytically active proteins were found to catalyze the progress of various physiological activities (Figure S2c).

**Figure 6:**
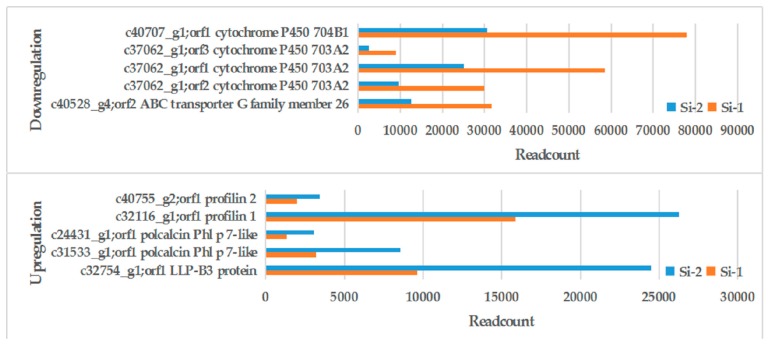
Histogram of differentially expressed proteins (DEPs). Blue column stands for Si-2, red column stands for Si-1. DEGs expression were expressed by readcount.

Based on the DEGs screened by the transcriptome, we focused on the corresponding DEPs in the proteome (Figure 6). The protein related to the formation of the pollen outer wall had a high expression in the Si-1 period. The allergic proteins increased significantly during the formation of the outer wall and were highly expressed in the Si-2 period. Pollen contains a lot of allergic proteins in the mature pollen stage.

### 2.5. Gene Cloning and Protein Subcellular Localization

The seven DEGs in Table 3 belonged to three major allergen families, among which the profilin family and the polcalcin family are both pollen pan-allergens. We aligned the three profilin family proteins, c32116_g1, c26567_g2, and c40755_g2, with the profilin allergens of other species, and building a phylogenetic tree by the neighbor-joining (NJ) method (Figure S3a). The result showed that c32116_g1 had the highest homology with other allergens. Two polcalcin family proteins c24431_g1 and c31533_g1 were aligned with other polcalcin allergens (Figure S3b). The result showed that c24431_g1 had the highest homology with other polcalcin allergens. We speculated that c32116_g1 and c24431_g1 were potential lily pollen allergens for testing in subsequent experiments.

Two genes, c32116_g1 and c24431_g1, were isolated from ‘Siberia’ by rapid amplification of cDNA ends (RACE) PCR, and were named *LoProfilin* and *LoPolcalcin* (Figure S4). Among them, the open reading frame length of *LoProfilin* was 396 bp, which was predicated to encode 131 amino acids. The open reading frame length of *LoPolcalcin* was 246 bp, which was predicated to encode 81 amino acids.

**Figure 7:**
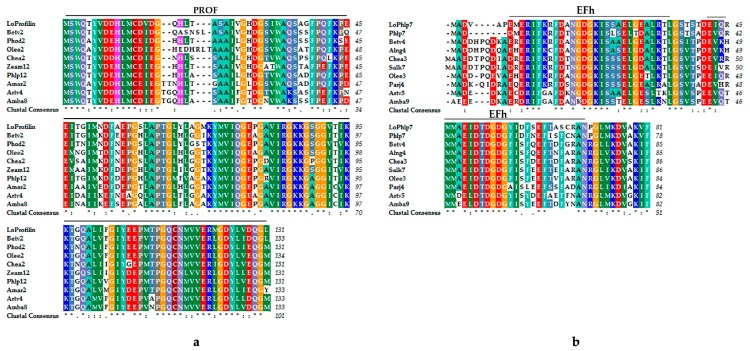
Protein sequence alignment of lily. (**a**) Profilin allergens of various species, Beta v 2, *Beta vulgaris*; Pho d 2, *Phoenix dactylifera*; Ole e 2, *Olea europaea*; Che a 2, *Chenopodium album*; Zea m 12, *Zea mays*; Phl p 12, *Phleum pratense*; Ama r 2, *Amaranthus retroflexus*; Art v 4, *Artemisia vulgaris*; Amb a 8, *Ambrosia artemisiifolia*. (**b**) Polcalcin allergens of various species, Phl p 7, *Phleum pratense*; Bet v 4, *Betula verrucosa*; Aln g 4, *Alnus glutinosa*; Che a 3, *Chenopodium album*; Sal k 7, *Salsola kali*; Ole e 3, *Olea europaea*; Par j 4, *Parietaria judaica*; Art v 5, *Artemisia vulgaris*; Amb a 9, *Ambrosia artemisiifolia*. ‘*’ represented completely identical amino acids; ‘:’ represented amino acids of the same polarity; ‘.’ represented differential amino acids.

The amino acid sequences of various allergens were queried through the allergen database. LoProfilin had a conserved PROF domain. Protein homology and phylogenesis analysis revealed that the profilin family protein sequences were extremely conserved (Figure 7a). Aligning the LoPolcalcin sequence with other polcalcin allergen sequences, LoPolcalcin was found to have two EF-hand domains (Figure 7b).

**Figure 8:**
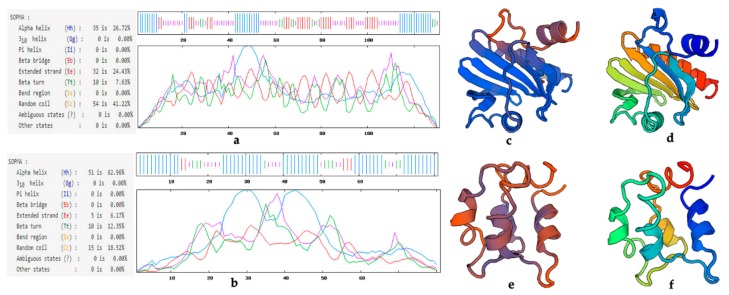
The predicted secondary structure tertiary structure of LoProfilin and LoPolcalcin. (**a**) LoProfilin secondary structure prediction. (**b**) LoPolcalcin secondary structure prediction. (**c**) LoProfilin tertiary structure prediction. (**d**) Bet v 2 tertiary structure. (**e**) LoPolcalcin tertiary structure prediction. (**f**) Phl p 7 tertiary structure prediction.

The primary structures of LoProfilin and LoPolcalcin proteins were very conserved, and we then analyzed their secondary structure (Figure 8). The results showed that the secondary structure of LoProfilin contained 26.72% alpha helix, 24.43% extended strand, 7.63% beta turn, and 41.22% random coil, while LoPolcalcin contained 62.96% alpha helix, 6.17% extended strand, 12.35% beta turn, and 18.52% random coil. We predicted the tertiary structure of LoProfilin and LoPolcalcin using homology modeling. The similarity of their three-dimensional structure can be seen in Figure 8. The structural coverage of LoProfilin (c) and birch pollen allergen Bet v 2 (d) was 99%. The structural coverage of LoPolcalcin (e) and timothy pollen allergen Phl p 7 (f) was 95%.

**Figure 9:**
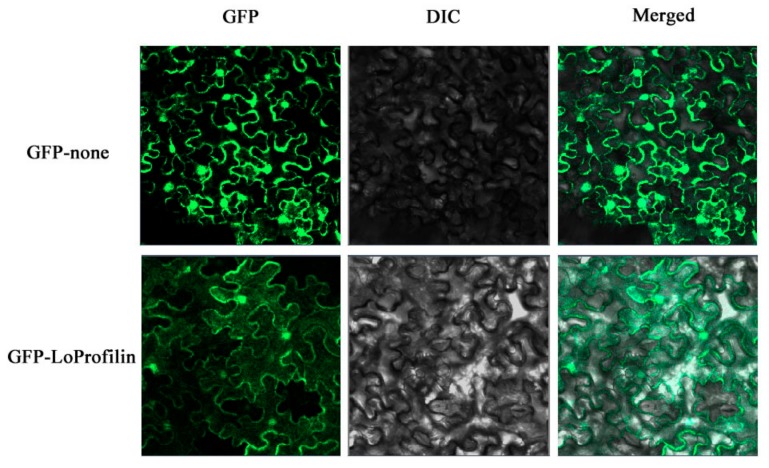
Profilin protein subcellular localization. From right to left, the figure shows dark, bright, and merged fields. Bar = 50 um.

We constructed the *35S::LoProfilin-GFP* plasmid, and transformed it into tobacco leaves by *Agrobacterium* infection. The control was a *35S::GFP* plasmid. The result of subcellular localization showed that the GFP fluorescences of LoProfilin-GFP were distributed in all areas of tobacco leaf cells, which indicated that LoProfilin localized in the entire tobacco cell (Figure 9).

### 2.6. Expression Analysis of Allergic Related Genes at Different Developmental Stages

**Figure 10:**
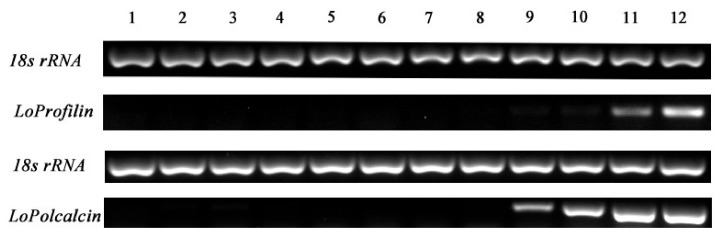
Expression analysis of allergic protein genes during pollen development. (1) Pollen of a 30 mm flower bud. (2) Pollen of a 40 mm flower bud. (3) Pollen of a 50 mm flower bud. (4) Pollen of a 54 mm flower bud. (5) Pollen of a 60 mm flower bud. (6) Pollen of a 70 mm flower bud. (7) Pollen of an 80 mm flower bud. (8) Pollen of a 90 mm flower bud. (9) Pollen of a 100 mm flower bud. (10) Pollen of a 110 mm flower bud. (11) Pollen from a just cracked anther. (12) Loose pollen.

An RT-PCR was used to detect the expression of two major allergenic genes in all 12 stages of pollen development (Figure 10). With the development of pollen, the expression of *LoProfilin* (c32116_g1) and *LoPolcalcin* (c24431_g1) displayed an increasing trend. The highest expression occurred at the loose powder stage. This result indicated that pollen allergic protein genes (*LoProfilin* and *LoPolcalcin*) expression increased during pollen maturation.

## 3. Discussion

Pollen plays an important role in plant reproduction [28]. The formation of pollen walls is the basis for the normal development of pollen, with the abnormal development of pollen walls leading to pollen abortion [29]. The pollen wall has an important protective effect on pollen [30]. At the same time, the structure of the pollen wall is also important for the recognition of pollen and stigma [31]. Pollen can also generate harmful effects. During the pollination period, pollen is scattered widely and can cause serious pollen pollution problems. One such pollen pollution problem is an anaphylactic reaction, which is caused by pollen wall proteins. The outer wall of the pollen is enriched with a variety of allergenic proteins, causing allergies in some people [9]. In recent years, an increasing number of people have been reported to suffer from pollen allergies, with the incidence rate in some areas of China reaching 5% [32]. Studies of the allergens in the lily pollen wall are therefore of great significance for solving problems such as the purification and identification of allergens.

### 3.1. Allergenic Protein Sequence Structure Analysis

All of the allergens identified were included in the allergen database (http://www.allergen.org/), with profilin and polcalcin being the main families of plant panallergens. Profilin from different species is regarded as a group of ‘panallergens’ because they share common IgE-binding epitopes [14]. Compared to the profilin of other species, the structure of the LoProfilin protein is very conservative. Although the secondary structure of the different profilins was different, the tertiary structure was very similar, with the conserved structure providing the binding site of lgE. Beta v 2 belongs to the profilin family of proteins. Its amino acid sequence is extremely short, but still exhibits allergic properties [33]. Its amino acid sequence may contain a conserved LgE binding site. It has been reported that the expression of a heterologous plant profilin in profilin-deficient dictyostelium cells can rescue the aberrant phenotype [34]. Profilin may have similar functions in different species due to its conserved structure. Many pollen-specific profilins have been identified as allergens, e.g., Ole e 2, Bet v 2, Amb a 8, and Art v 4 [35,36]. A profilin gene *BnPFN* is a pollen-specific gene that researchers have suggested is the main cause of pollen anaphylactic reactions [37]. In our study, the LoProfilin protein that was highly expressed in mature pollen was screened and aligned. We suspect that LoProfilin may be one of the lily pollen allergens.

Phl p 7 is an allergenic protein derived from *Phleum pratense* pollen. It is a 2-EF arm calcium-binding protein, with two EF-hand domains [16]. Phl p 7 contains two lgE binding sites and its three-dimensional structure forms a barrel dimer to combine with LgE [38]. The amino acid sequences of LoPolcalcin and Phl p 7 were very similar. The three-dimensional structure of LoPolcalcin needs further study. Due to the conservation of these protein sequences and functions, they may have allergic properties in lily pollen. The analysis and prediction of lily allergens required a cloning and sequence analysis of *LoProfilin* and *LoPolcalcin*, which laid the foundation for our isolation and purification of lily allergens, and provided an important reference for the identification of subsequent lily allergens.

### 3.2. Profilin Proteins in Pollen Wall

LoProfilin is highly expressed in mature pollen at both the transcriptional and protein levels. Profilin proteins have been identified as pollen allergens in other species. AtPRF4 protein is highly expressed in *Arabidopsis thaliana* mature pollen and is not expressed in other vegetative tissues [39]. Three profilins of maize have been also reported, which are specifically expressed in pollen or anther [40]. In tobacco, the profilin protein is increased with the pollen development, and the expression of profilin in mature pollen is 50–100-fold higher than that of other tissues [41]. These profilin were expressed significantly more in mature pollen than other tissues or expressed specifically in pollen and anthers, which suggests they may play an important role in pollen development and plant reproduction.

Profilin is an actin-binding protein, and an important assembly factor in actin assembly [42]. In addition, the actin participates in the composition of cytoskeleton [43]. The distribution of cytoskeleton in cells is extensive, and the cytoskeleton includes the nuclear skeleton, cytoplasmic skeleton, cell membrane skeleton, and extracellular matrix [44]. A research in Arabidopsis has reported that both constitutive and pollen-specific profilins are abundant in the cytoplasm and nucleus [45]. However, the nuclear or other localization of profilin had also been reported in birch and maize [46,47]. Therefore, the distribution of profilin in cells is extensive and plays an important role in the formation of cytoskeleton and maintenance of cell structure. Our results showed that LoProfilin was localized in the entire tobacco cell, which implies the similar function of Loprofilin for cell structure.

## 4. Materials and Methods

### 4.1. Experimental Materials

Lily was planted in an experimental field at Nanjing Agricultural University (located at 31°82′ north latitude and 118°66′ east longitude), Nanjing, Jiangsu Province, China. The lily cultivar Lilium Oriental Hybrids ‘Siberia’ was used in this experiment.

We collected 45 lily flower buds (size, 56–61 mm). One flower bud contained six anthers. All anthers were in the same developmental stage. One anther was used to observe the pollen development period, then we extruded the pollen from the other five anthers as a sample. Eighteen samples in the early mononuclear stage were selected, named Si-1. Six samples were mixed into one biological replicate and Si-1 had three biological replicates. When the anther was cracked, one anther was used to observe the pollen development period, and we took pollen from the other five anthers as a sample. Eighteen samples of mature pollen were selected, named Si-2. Six samples were mixed into one biological replicate and Si-2 had three biological replicates.

### 4.2. Histological Observation and Sample Collection

Flower buds of different sizes were collected, and the anthers were removed. The anthers were cut and squeezed to distribute the pollen evenly on a microscope slide, and were then stained by improved phenol magenta and observed under an optical election microscope (DM 6B, Leica, Wetzlar, Germany). Mononuclear early pollen (Si-1) and mature pollen (Si-2) were collected in the mononuclear and loose powder stages, respectively. Samples were immediately frozen in liquid nitrogen and stored at −80 °C. Three biological replicates were prepared from each sample.

### 4.3. RNA Extraction and Quality Determination

The total RNA of six samples in two periods (three repetitions of each period) were extracted by using the trizol method (Takara Bio Inc., Otsu, Japan). Gel electrophoresis was used to detect whether rna was degraded or contaminated. We use a spectrophotometer to detect rna purity (Implen, Westlake Village, CA, USA). Then, we used Qubit^®^ RNA Assay Kits (Life Technologies, Carlsbad, CA, USA) and an RNA Nano 6000 Assay Kit (Agilent Technologies, Santa Clara, CA, USA) to test the RNA concentration and integrity, respectively.

### 4.4. Library Preparation and Transcriptome Sequencing

We uesd the RNA of six samples from Si-1 and Si-2 as material, and enriched the mRNA with magnetic beads. Pyrolysis was applied to convert them into short fragments in a buffer. Random hexamer and M-MuLV enzyme (Takara Bio Inc., Otsu, Japan) were used to synthesize first strand cDNA. DNA Polymerase I and RNase H (Takara Bio Inc., Otsu, Japan) were used to synthesize the second strand cDNA. EB buffer was used for end repair and for the addition of adenine (A), with the sequencing linker and A-tailed fragment and amplification sequence linked by PCR. Finally, we constructed six cDNA libraries.

According to the manufacturer’s instructions, the six library preparations were sequenced on an Illumina Hiseq platform in the Novogene Experimental Department.

### 4.5. Transcriptome Assembly and Functional Annotation

Samples of Si-1 (three biological replicates) and Si-2 (three biological replicates) were used to construct six cDNA libraries. All sequencing results were performed using double-ended sequencing. Then, all sequencing data were integrated by the Trinity method [48]. We uploaded the RNA-Seq data to the National Center for Biotechnology Information (NCBI) (SRA submission: SUB6179923).

We annotated gene functions based on seven large databases: NCBI, Nr, Nt, Pfam, KOG/COG, Swiss-Prot, KEGG Ortholog, and GO.

### 4.6. Gene Differential Expression Analysis

The input data of differential expression of genes are the readcount data. We averaged the normative readcount from three biological replicates. The log2FC was log2 (Si-2 readcount/Si-1 readcount). Differential expression analysis of two groups was performed using the DESeq R package (1.10.1). DESeq provide statistical routines for determining differential expression in digital gene expression data using a model based on the negative binomial distribution. The resulting *p* values were adjusted using the Benjamini and Hochberg’s approach for controlling the false discovery rate. Genes with an adjusted *p*-value < 0.05 found by DESeq were assigned as differentially expressed.

### 4.7. Quantitative Real-time Polymerase Chain Reaction (qRT-PCR) Analysis

A qRT-PCR analysis was used to verify the expression levels of genes identified in RNA sequencing. Si-1 and Si-2 were performed with three biological replicates and three technical replicates. Real-time PCR was performed with specific primers that were designed based on the selected unigene sequences with Primer express (Primer premier 6.0, Canada) software (Table S1). The 18S rRNA gene was used as the reference sequence for quantitative expression analysis. The qRT-PCR assays were conducted as described by Dekkers et al. [49].

### 4.8. Total Protein Extraction

Six samples of Si-1 (three biological replicates) and Si-2 (three biological replicates) were milled individually with liquid nitrogen. Then, SDS (0.2%), urea (8M), and 50 mM Tris-HCl buffer (pH = 8) were added. The samples were mixed together and incubated. They were sonicated on ice for 5 min, and centrifuged at 12,000× rpm, for 15 min at 4 °C. The protein concentration in the supernatant was determined, DTT (2 mM) was left at 56 °C for 1 h. Then, four volumes of cold acetone were added and the solutions were vortexed at −20 °C for 2–8 h. The collected precipitate was washed with cold acetone. TEAB (0.1 M, pH = 8.5) and urea (8 M) were used to dissolve the precipitate. Finally, the protein concentration was determined.

### 4.9. Functional Annotation and Database Analysis

The functional annotation of identified proteins was completed using the GO, KEGG, and COG databases. Domain annotations (IPR) were performed using interproscan software, including the databases of domains such as Pfam, ProDom, and SMART. Domain annotations for functionally unknown proteins were determined using pattern structures or features.

### 4.10. Isolation of LoProfilin and LoPolcalcin from Lily

Specific primers (LoPro and LoPol, Table S1) were designed based on transcriptome sequences and the coding sequence of *LoProfilin* and *LoPolcalcin* using PrimeSTAR HS DNA polymerase (Takara Bio Inc., Otsu, Japan). The final PCR procedure was: 98 °C for 5 min, 98 °C for 10 s, 59 °C for 15 s, 72 °C for 30 s (35 cycles), and 72 °C for 3 min. The PCR products were tested by 1% agarose gel electrophoresis, and the target DNA band was recovered using a midi purification kit. The target fragment was constructed into a pMD18-T vector, and transformed into competent cells of Escherichia coli strain DH5a for sequencing. Alignment sequencing results were obtained using DNAMAN (DNAMAN 6.0.3.40, USA) sequence analysis software.

### 4.11. Protein Structure Analysis

The sequences of identified allergens were obtained from the Allergen Nomenclature database (http://www.allergen.org/). An analysis of amino acid sequence similarity was conducted using Clustalx (ClustalX 1.81, Ireland) and BioEdit (BioEdit version 7.0.5.3, Japan) software. The prediction of protein secondary and tertiary structure was conducted using the self-optimized prediction method with alignment (SOPMA) (https://npsa-prabi.ibcp.fr/cgi-bin/npsa_automat.pl?page=npsa_sopma.html) and SWISS-MODEL (https://swissmodel.expasy.org/interactive), respectively.

### 4.12. Subcellular Localization of LoProfilin Protein

The construction of *LoProfilin* into the pCAMBIA1300-GFP vector was conducted by homologous recombination. The recombinant plasmid was transformed into competent cells of *Agrobacterium* strain GV3101. Bacterial liquid was injected into tobacco cells. Tobacco plants were placed in the dark for one day and in the light for one day. The green fluorescent protein (GFP) signal was observed with a laser scanning confocal microscope.

### 4.13. Reverse Transcriptase PCR

An RT-PCR was used to detect the expression of allergic protein-related genes in the different developmental stages of pollen. Pollen samples were collected from the sporulation stage to the mature pollen stage. The length of the anther was sampled at 30, 40, 50, 56, 60, 70, 80, 90, 100, and 110 mm, respectively. These samples were numbered 1–10. When the flower opened, as the anther cracked its pollen was sampled (sample number 11). When the anther was completely cracked its pollen was also sampled (sample number 12). Samples were subjected to rapid freezing in liquid nitrogen, RNA was extracted by the Trizol method, and specific quantitative primers were designed to detect changes in the expression of the target genes.

## 5. Conclusions

In this study, a systematic study was carried out to investigate the cytological characteristics of pollen formation in *Lilium* oriental hybrid ‘Siberia’. The analysis of the pollen transcriptome identified DEGs, e.g., *Profilin*, *Phl p 7 (Polcalcin)*, *Ole e 1*, and *Phl p 11*, which were associated with pollen allergens. The results of pollen proteome analysis positively verified a significant increase of the content of LoProfiilin and LoPolcalcin, both of which were annotated as allergen proteins. Then, the *LoProfiilin* and *LoPolcalcin* were isolated and cloned from lily; the protein structural prediction and gene expression analysis of them identified that LoProfilin and LoPolcalcin as potential allergens in lily pollen. Overall, this study would provide valuable information for searching potential allergen of lily pollen to solve the problem of pollen allergy.

## Figures and Tables

**Table 1:** Comparison of the lily pollen development process and floral organ morphological characteristics.

Pollen Development Stage	Size of Bud (mm)	Size of Anther (mm)	Coler of Anther	Color of Pollen
Sporulation stage	<36	<17	Light green	Transparent
Mother cell stage	36–43	17–24	Light yellow	Light yellow
Meiosis stage	43–53	24–29	Light yellow	Light yellow
Tetrad stage	54–56	29–32	Light yellow	Light yellow
Early mononuclear stage	56–61	32–35	Yellow	Yellow/Dark yellow
Mature pollen stage	>61	>35	Orange	Orange

**Table 2:** Comparison of lily pollen development process and floral organ morphological characteristics.

Sample Name	Raw Reads	Clean Reads	Clean Bases	Q30 ^1^	Clean Reads Ratio	GC ^2^	Total Number of Unigenes	Total Length of Unigenes (bp)
Si-1	65,663,717	62,398,416	9.36	92.56	95.03	50.81		62,531,354
Si-2	67,241,165	64,561,039	9.68	92.85	96.01	51.72		

^1^ The percentage of bases in which quality was greater than 30. ^2^ The percentage of G and C bases in all transcripts.

**Table 3:** Differentially expressed genes (DEGs) involved in lily pollen wall formation and allergens.

Gene ID	Si-1 Readcount ^1^	Si-2 Readcount ^2^	Log2FC ^3^ (Si-2 vs Si-1)	Up/Down	Annotation
c40528_g4	4272.08	18.92	−7.82	Down	ABC transporter G family member 26
c38800_g1	2816.89	64.91	−5.44	Down	transcription factor GAMYB isoform X2
c38779_g1	2810.16	1233.06	−1.19	Down	transcription factor GAMYB-like isoform X3
c37062_g1	8832.13	0	-Inf	Down	cytochrome P450 703A2
c30037_g1	9.48	0	-Inf	Down	cytochrome P450 703A2
c40707_g1	6459.93	0	-Inf	Down	cytochrome P450 704B1
c32754_g1	2.98	69,570.06	14.51	Up	Zea mays pollen allergen Phl p 11
c30725_g1	28.06	116.33	2.05	Up	pollen Ole e 1 allergen and extensin family protein
c31533_g1	1.68	27,325.19	13.99	Up	polcalcin Phl p 7-like
c24431_g1	0	39.70	Inf	Up	polcalcin Phl p 7-like
c32116_g1	6.17	22,833.04	11.85	Up	Profilin-1
c26567_g2	995.57	11,259.00	3.50	Up	Profilin-3
c40755_g2	870.39	1572.53	0.85	Up	Profilin-2

^1^ The average of the normative readcounts from Si-1 three samples. ^2^ The average of the normative readcounts from Si-2 three samples. ^3^ log2(Si-2 readcount/Si-1 readcount).

## Supplementary Materials

Supplementary materials can be found at https://www.mdpi.com/1422-0067/20/23/5892/s1.

## Abbreviations

DEG	Differentially expressed gene
COG	Cluster of Orthologous Groups
KEGG	Kyoto Encyclopedia of Genes and Genomes
GO	Gene Ontology
TMT	Tandem mass tag
PPI	Protein-protein interaction
