# Supplementary material for: Analysis of Pollen Allergens in Lily by Transcriptome and Proteome Data
Source: Int J Mol Sci. 2019 Nov 24;20(23):5892. doi: 10.3390/ijms20235892 (PMC6929097; doi:10.3390/ijms20235892)
Supplement: Supplementary file 1 [file ijms-20-05892-s001.zip › Supplementary materials/CWS_Editorial_Certificate.pdf]

Charlesworth Author Services

EDITORIAL CERTIFICATE

This document certifies that the manuscript below was edited for correct English language usage, grammar, punctuation and spelling by qualified native English speaking editors at Charlesworth Author Services.

Paper Title:

Analysis of pollen allergens by transcriptome and proteome in lily

Author:

泽 吴

Date certificate issued:

November 15, 2019

cwauthors.com
