# Supplementary material for: Analysis of Pollen Allergens in Lily by Transcriptome and Proteome Data
Source: Int J Mol Sci. 2019 Nov 24;20(23):5892. doi: 10.3390/ijms20235892 (PMC6929097; doi:10.3390/ijms20235892)
Supplement: Supplementary file 1 [file ijms-20-05892-s001.zip › Supplementary materials/Supplementary Figures.pptx]

## Slide 1
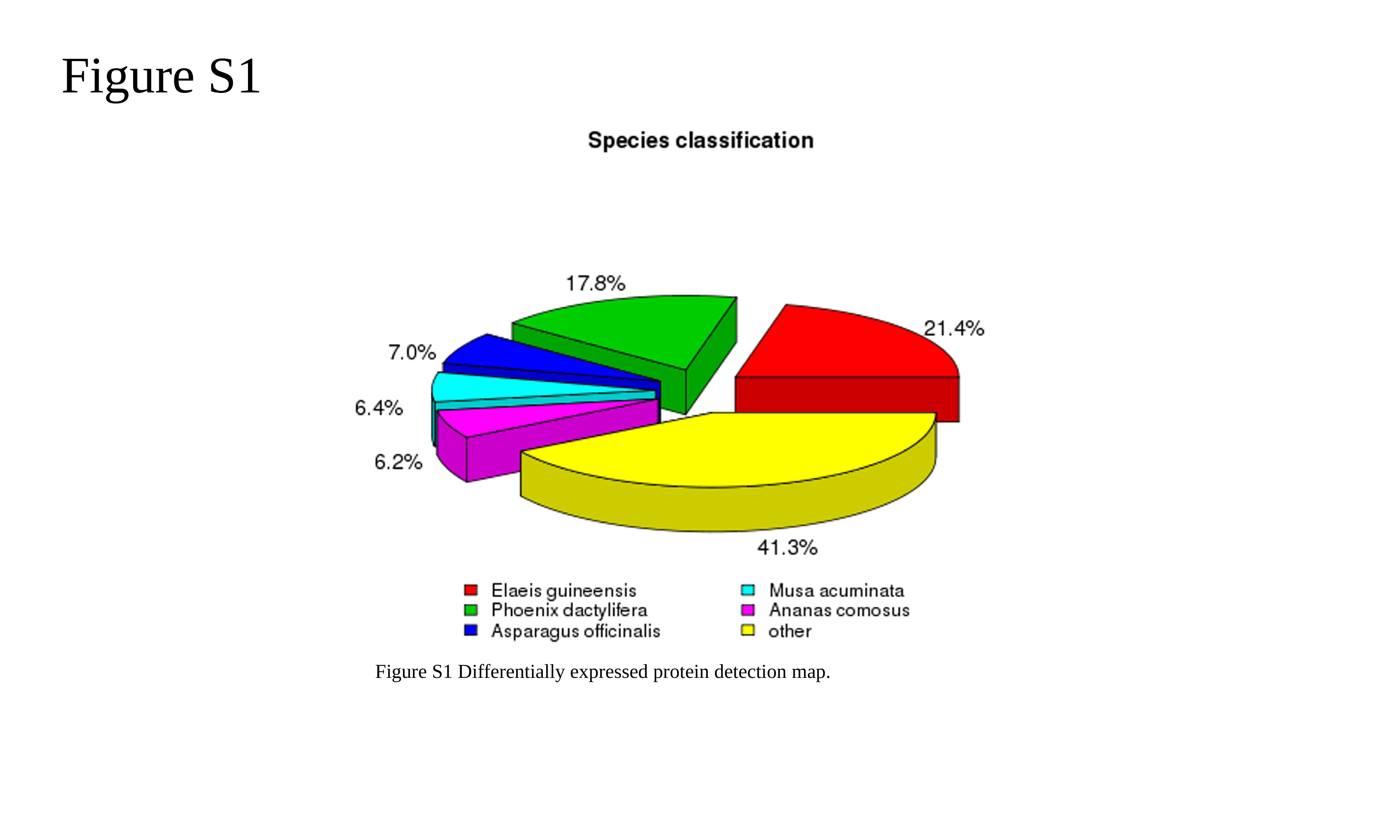

Figure S1
Figure S1 Differentially expressed protein detection map.

## Slide 2
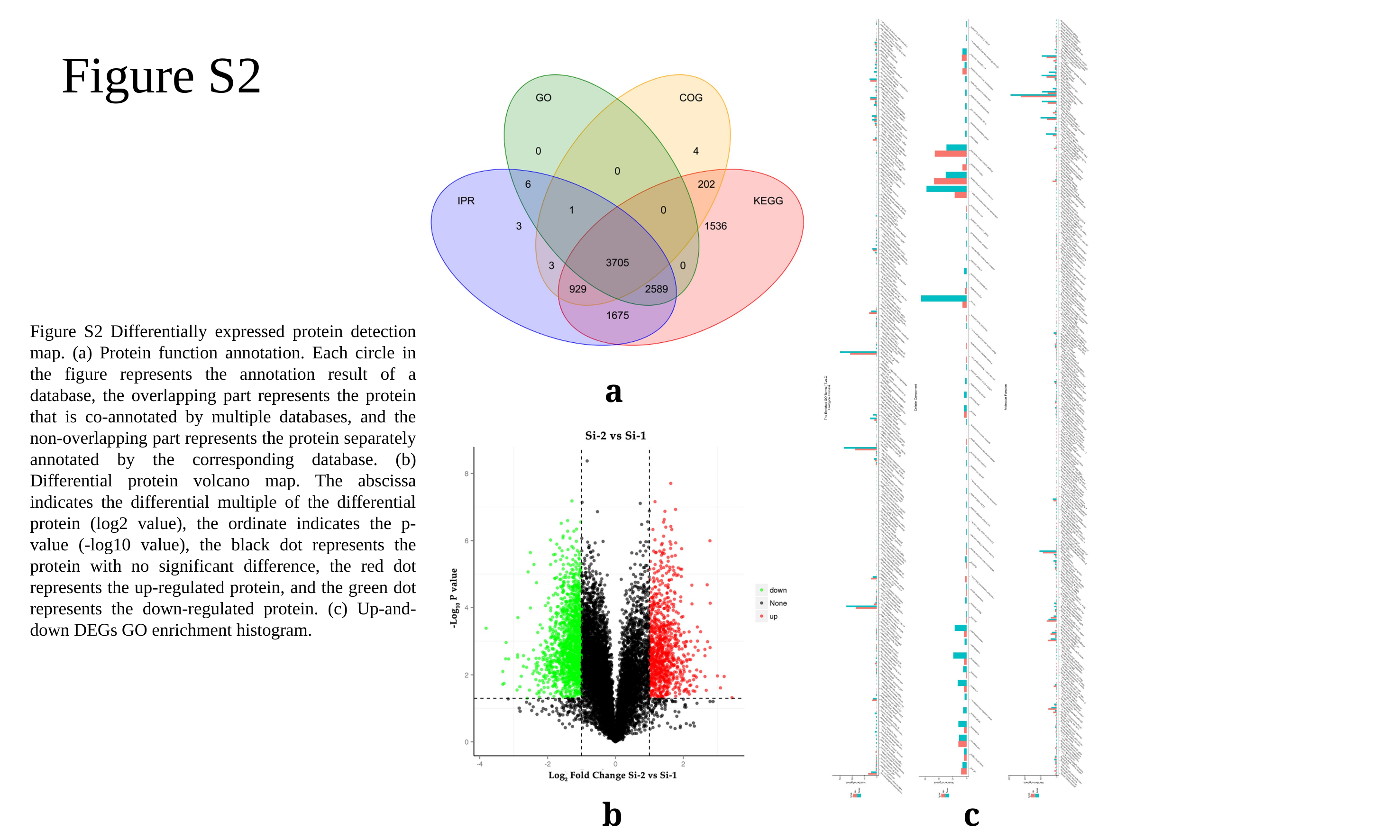

Figure S2
Figure S2 Differentially expressed protein detection map. (a) Protein function annotation. Each circle in the figure represents the annotation result of a database, the overlapping part represents the protein that is co-annotated by multiple databases, and the non-overlapping part represents the protein separately annotated by the corresponding database. (b) Differential protein volcano map. The abscissa indicates the differential multiple of the differential protein (log2 value), the ordinate indicates the p-value (-log10 value), the black dot represents the protein with no significant difference, the red dot represents the up-regulated protein, and the green dot represents the down-regulated protein. (c) Up-and-down DEGs GO enrichment histogram.
a
b
c

## Slide 3
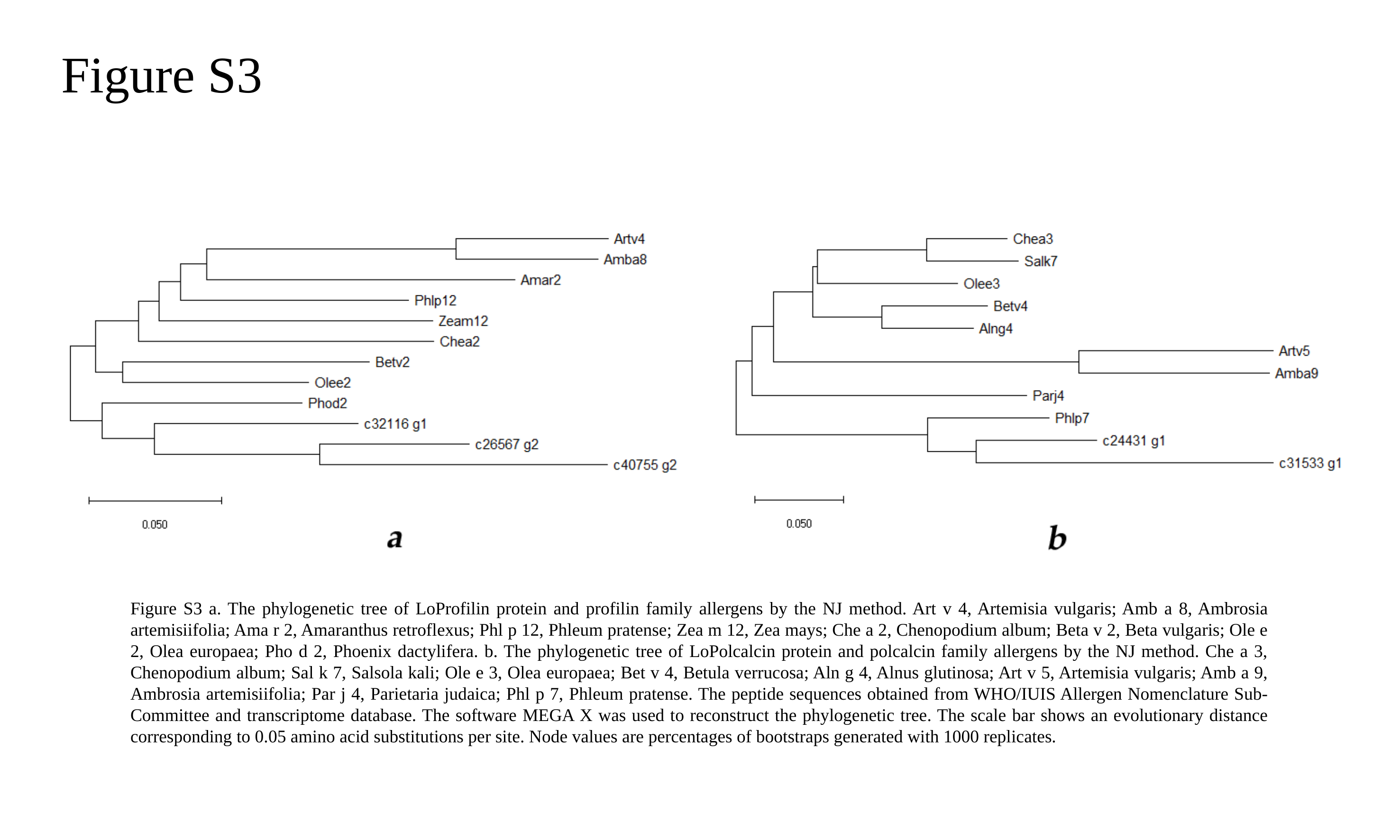

Figure S3
Figure S3 a. The phylogenetic tree of LoProfilin protein and profilin family allergens by the NJ method. Art v 4, Artemisia vulgaris; Amb a 8, Ambrosia artemisiifolia; Ama r 2, Amaranthus retroflexus; Phl p 12, Phleum pratense; Zea m 12, Zea mays; Che a 2, Chenopodium album; Beta v 2, Beta vulgaris; Ole e 2, Olea europaea; Pho d 2, Phoenix dactylifera. b. The phylogenetic tree of LoPolcalcin protein and polcalcin family allergens by the NJ method. Che a 3, Chenopodium album; Sal k 7, Salsola kali; Ole e 3, Olea europaea; Bet v 4, Betula verrucosa; Aln g 4, Alnus glutinosa; Art v 5, Artemisia vulgaris; Amb a 9, Ambrosia artemisiifolia; Par j 4, Parietaria judaica; Phl p 7, Phleum pratense. The peptide sequences obtained from WHO/IUIS Allergen Nomenclature Sub-Committee and transcriptome database. The software MEGA X was used to reconstruct the phylogenetic tree. The scale bar shows an evolutionary distance corresponding to 0.05 amino acid substitutions per site. Node values are percentages of bootstraps generated with 1000 replicates.

## Slide 4
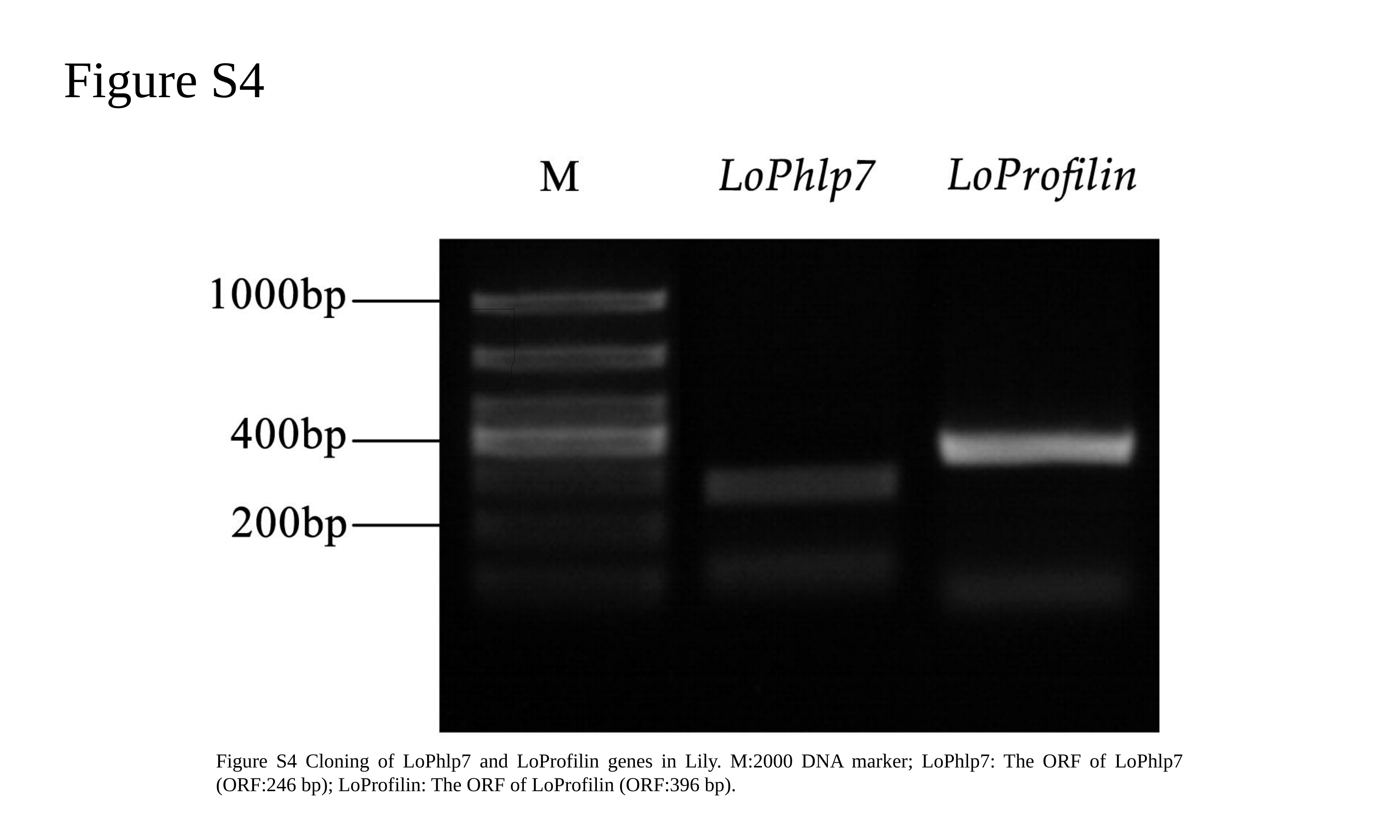

# Figure S4
Figure S4 Cloning of LoPhlp7 and LoProfilin genes in Lily. M:2000 DNA marker; LoPhlp7: The ORF of LoPhlp7 (ORF:246 bp); LoProfilin: The ORF of LoProfilin (ORF:396 bp).
